# Responses of the Pheromone-Binding Protein of the Silk Moth *Bombyx mori* on a Graphene Biosensor Match Binding Constants in Solution

**DOI:** 10.3390/s21020499

**Published:** 2021-01-12

**Authors:** Caroline Bonazza, Jiao Zhu, Roger Hasler, Rosa Mastrogiacomo, Paolo Pelosi, Wolfgang Knoll

**Affiliations:** 1 CEST Competence Centre for Electrochemical Surface Technology, 2700 Wiener Neustadt, Austria; caroline.kotlowski@boku.ac.at; 2 Austrian Institute of Technology GmbH, Biosensor Technologies, 3430 Tulln, Austria; jiao.zhu@ait.ac.at (J.Z.); roger.hasler@ait.ac.at (R.H.); paolo.pelosi@ait.ac.at (P.P.); 3 Department of Agriculture, Food, and Environment, University of Pisa, 56124 Pisa, Italy; rosa.mastrogiacomo@libero.it

**Keywords:** biosensor, field-effect transistor, graphene, odorant-binding protein, global analysis, fluorescence displacement assay, *Bombyx mori*

## Abstract

An electronic biosensor for odors was assembled by immobilizing the silk moth *Bombyx mori* pheromone binding protein (BmorPBP1) on a reduced graphene oxide surface of a field-effect transistor. At physiological pH, the sensor detects the *B. mori* pheromones, bombykol and bombykal, with good affinity and specificity. Among the other odorants tested, only eugenol elicited a strong signal, while terpenoids and other odorants (linalool, geraniol, isoamyl acetate, and 2-isobutyl-3-methoxypyrazine) produced only very weak responses. Parallel binding assays were performed with the same protein and the same ligands, using the common fluorescence approach adopted for similar proteins. The results are in good agreement with the sensor’s responses: bombykol and bombykal, together with eugenol, proved to be strong ligands, while the other compounds showed only poor affinity. When tested at pH 4, the protein failed to bind bombykol both in solution and when immobilized on the sensor. This result further indicates that the BmorPBP1 retains its full activity when immobilized on a surface, including the conformational change observed in acidic conditions. The good agreement between fluorescence assays and sensor responses suggests that ligand-binding assays in solution can be used to screen mutants of a binding protein when selecting the best form to be immobilized on a biosensor.

## 1. Introduction

Recently, odorant-binding proteins (OBPs), both from insects [1,2,3] and from mammals [4,5], have received increasing attention as biosensing elements for detecting odorants and pheromones in the environment [6,7,8]. OBPs are particularly suitable to such tasks thanks to special properties and characteristics:

1. Their exceptional stability to temperature, up to around 70 °C, allows for their use in demanding environmental conditions. They can also be denatured by chemicals and easily refolded back into their active conformations after removing the denaturing agent [9,10].
2. Being the natural carriers for odorant and pheromones, with dissociation constants in the micromolar range, they represent the best candidates to trap and detect environmental odors.
3. OBPs can be expressed in high yields in bacterial and eucaryotic systems and can be easily purified using standard protocols [11].
4. Based on the large amount of structural information [12,13], site-specific mutagenesis is easy to design with great confidence of success. This allows for the production of proteins tuned to the desired volatiles to monitor, when a specific OBP is not known to be present in nature [14,15,16].
5. Currently, the affinity of a ligand for an OBP is evaluated using a displacement assay, where a fluorescent probe is adopted as reporter [6]. This method is fast and simple, allowing for a wide screening of ligands. However, it suffers for being an indirect approach, and sometimes different fluorescent reporters can yield different affinity values for the same ligand [17]. Therefore, a label-free approach, such as the use of an electronic biosensor, would be highly desirable.

Generally, in many sensor formats, the protein is immobilized on the surface of a transducer, and changes in optical, electrical, or other parameters are measured upon interactions with volatile compounds. Optical sensors monitor changes in refractive index when a ligand binds to a protein receptor or a DNA target hybridizes to a capture probe immobilized on the transducer surface [18]. A particularly sensitive, and hence attractive, method based on surface plasmon resonance (SPR) is adopted in many commercial instruments. It has been applied with success in numerous cases to detect interactions between two proteins, such as those between antigen and antibody [19], between proteins and their specific aptamers [20], or between two (relatively) large fragments of DNA [21].

If the ligand to be monitored is a small molecule, the sensitivity is an issue because the binding to its receptor changes the overall optical architecture only to a minute degree. Still, in some cases, this approach has been reported to detect binding of odorants to olfactory receptors [22,23]. In any case, this technique requires large and expensive pieces of equipment.

In electronic smell sensors, an OBP is immobilized on the surface of a (semi-) conducting substrate, and changes in voltage, current, or impedance are monitored following the binding of odorants. Field-effect transistors with the pig OBP immobilized on gold [24] were used to monitor, with good sensitivity and selectivity, the interactions of the protein with some odorants. In these devices, the OBP-derivatized electrode acts as a liquid gate of the transistor. In other reports, the best odorants could be detected down to micromolar concentrations [25,26]. Alternatively, it was shown that the honeybee OBP14 could be covalently attached to reduced graphene oxide as the semi-conductive material, with the current between the source and the drain electrode being modified upon ligand binding to the OBP [27,28].

All these approaches have in common the need to immobilize the protein covalently to a solid surface. This is usually done by establishing bonds between ε-amino groups of lysine residues of the protein and activated carboxy groups on the surface of the sensor. Consequently, the protein is attached in random orientations, thus making the binding interactions rather unpredictable. Moreover, one cannot exclude the fact that the protein might behave differently in solution and when immobilized on a solid substrate. Therefore, we decided to compare the binding properties of an OBP measured in the classical fluorescence assay with the responses of a biosensor bearing the same protein immobilized on a surface of reduced graphene oxide. We focus on the use of graphene-based field-effect transistors, as they have been shown to give excellent sensing performance parameters in terms of high specific surface area, excellent electronic properties, and high flexibility [29].

For this task, we chose the *Bombyx mori* pheromone-binding protein (PBP1), the first insect OBP to be crystallized [30]. This protein was shown to bind, with similar affinities, the two main pheromones of the silk moth, bombykol and bombykal [31]. Therefore, we selected these two pheromones, as well as some general odorants of unrelated structure, to compare the behavior of the protein in the two different approaches. Moreover, we also measured the binding of bombykol at pH 4, where a major conformational change has been reported to occur, strongly reducing the affinity of the protein for its ligands [32].

## 2. Materials and Methods

### 2.1. Protein Expression and Purification

**Figure 1:**
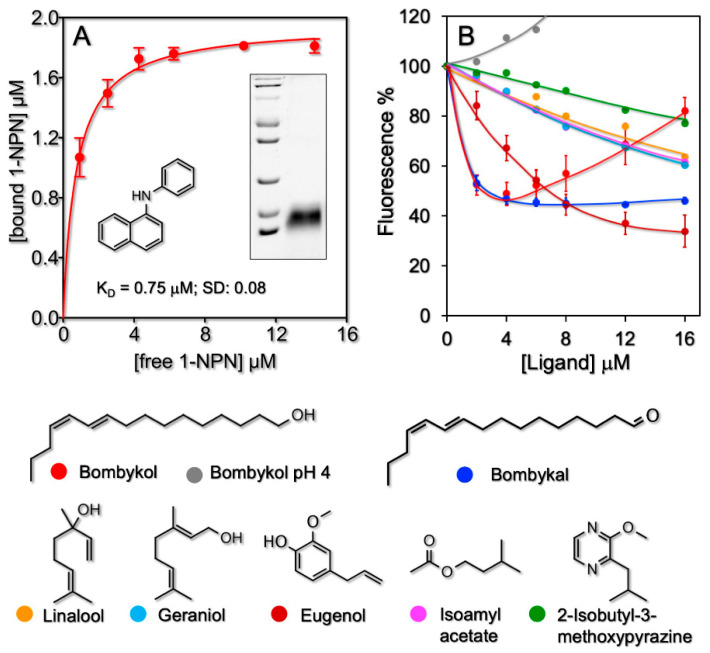
Binding activity of *B. mori* pheromone-binding protein (PBP)1 measured in solution along with the fluorescent assay. (**A**) The protein binds, with good affinity (0.75 μM), the fluorescent probe N-phenyl-1-naphthylamine (1-NPN), whose structure is shown. The inset reports the electrophoretic analysis (SDS-PAGE) of a sample of the recombinant protein used in all experiments. (**B**) Displacement of the fluorescent probe 1-NPN by different chemicals. The protein and the probe, both at the concentration of 2 µM, were titrated with increasing amounts of each competitor. Bombykol and bombykal are the strongest ligand. Of the other compounds tested, only eugenol exhibited appreciable affinity. Interestingly, the affinity of bombykol is completely abolished at pH 4 due to a major conformational change of the protein [32]. The increasing behavior of the bombykol displacement curve at concentrations higher than 3–4 µM is due to micelles formation [6].

A synthetic gene encoding the mature sequence (with the sole addition of the initial methionine) of PBP1 of *B. mori* (acc. no. P34174) was subcloned in the expression vector pET15b (Novagen, Darmstadt, Germany), along with standard procedures, allowing the protein to be expressed as a fusion construct bearing a polyhistidine (his-tag) at the amino terminus. Following selection of the colonies containing the insert performed by PCR analysis, positive clones were used to express the protein in *Escherichia coli*, following a standard protocol. After sonication, the protein was mainly present in the pellet and was solubilized in 8 M urea and 1 mM dithiothreitol (DTT). Refolding was accomplished by extensive dialysis (three times overnight with changes of buffer) against 50 mM Tris-HCl, pH 7.4. The PBP1 was purified by affinity chromatography on Ni ion affinity chromatography (GE Healthcare) along with the manufacturer’s protocol. The purity of the protein was assessed by SDS-PAGE (inset of Figure 1A).

### 2.2. Fluorescence Binding Assay

Dissociation constants were measured in solution along with the widely adopted fluorescence binding assay [9,14]. Spectra were recorded on a PerkinElmer FL 6500 spectrofluorometer at room temperature in a right-angle configuration, with a 1-cm light path quartz cuvette and 5-nm slits for both excitation and emission.

First, the binding of BmorPBP1 to the fluorescent probe N-phenyl-1-naphthylamine (1-NPN) was measured by titrating a 2-μM solution of the protein in 50 mM Tris-Cl buffer at pH 7.4 with aliquots of 1 mM solution of 1-NPN in methanol to final concentrations of 2–16 μM. Excitation wavelength was 337 nm and intensity was recorded at the maximum of the emission peak, 408 nm. The affinities of other ligands were evaluated in competitive binding assays by titrating a solution of the protein and 1-NPN, both at the concentration of 2 μM, with aliquots of 1-mM methanol solutions of each chemical to final concentrations of 2–16 μM. The affinity to 1-NPN was calculated using Prism software. Dissociation constants of competing ligands were evaluated from the corresponding [IC]_50_ values (the concentration of each ligand halving the initial value of fluorescence), using the equation: K_d_ = [IC]_50_/1 + [1-NPN]/K_NPN_, where [1-NPN] is the concentration of free 1-NPN and K_NPN_ the dissociation constant of the complex Protein/1-NPN.

### 2.3. Fabrication of BmorPBP1 Based Biosensor Devices

Reduced graphene oxide field-effect transistors (rGO-FET) were fabricated as previously described [33] with a modified channel width of 30 µm. Electrical properties of the FET devices were tested along with the procedure [34]. For the detection of different odorants, the rGO-based biosensors were functionalized with BmorPBP1. Briefly, for the attachment of the protein to the sensing area, the graphene surface was chemically modified with a bifunctional linker: a 10-μL droplet of a 10-μM 6-(4-(pyren-3-yl) butanamido)-2-(bis(carboxymethyl) amino) hexanoic acid solution was applied overnight to the rGO channel area, rinsed, incubated for 30 min with a 40-mM NiCl_2_ solution (pH 5.5, adjusted with KOH), rinsed, then incubated with 20 μL of a 10-μM protein solution, followed by a final rinse.

All the steps for the fabrication of the odorant biosensor device, including successful reduction of GO to rGO, linker binding, and protein attachment, were carefully checked using spectroscopic methods [27].

### 2.4. Electrical Measurements

The evaluation of BmorPBP1 binding affinities to various odorants was performed under the following standardized conditions: electrical measurements were taken using a Keithley 4200 semiconductor characterization system. An Ag/AgCl reference electrode (Flex ref, World Precision Instruments) was used to operate the FET device in liquid gate configuration with a constant gate bias (V_g_) of −0.6 V and a constant source-drain bias (V_DS_) of 0.05 V. A flow rate of the odorant solutions in the flow cell was set at 300 µL/min in order to minimize mass transfer limitations of the analytes to the sensor surface [35,36,37]. The general procedure of the whole titration experiment started with continuously flushing the flow cell with pure buffer (1 mM PBS, pH = 8.0), until a stable baseline of source-drain current, I_DS_, was established. Subsequently, the biosensor was titrated with different odorant concentrations (1 μM–4 mM) and the change in source–drain current (ΔI_DS_) was monitored in real time. Here, the lowest odorant concentration was injected first, and the odorant was allowed to interact with the surface immobilized BmorPBP1 to reach equilibrium, as indicated by a constant source–drain current. This process was repeated with odorant solutions of higher concentrations, until the surface was fully saturated with the target analyte, as indicated by a constant source–drain current (I_DS_). Surface titration experiments were terminated by a final washing step using pure buffer in order to dissociate the formed PBP1-odorant complex, resulting in almost complete restoration of the initial baseline current. As observed in almost all biosensor devices, a slight drift in source–drain current was observed with time. For this reason, the drain current response curves were normalized by subtraction to the baseline current. Kinetic parameters, as well as the dissociation constant, K_d_, of the above described titration experiments were analyzed using the Langmuir model [38].

### 2.5. Analysis of Odorants Binding to BmorPBP1, Immobilized on rGO-FET

With the electronic transducer directly coupled to a flow cell, one can perform titrations as well as kinetic measurements by online changing of the analyte solution (pure buffer, different analytes at different concentrations) flowing through the cell, while recording the sensor response, i.e., a change in the source–drain current, ΔI_DS_ = I_DS_ − I_DSBaseline_, in real time [39].

Earlier studies have given evidence that the underlying molecular mechanism of the recognition and binding of odorant analytes from solution to the surface-immobilized protein, BmorPBP1 in this case, can be well described by the Langmuir adsorption model [38]: the sensor has a fixed number of binding sites on its surface, whose occupancy is measured by Θ, the coverage, ranging from 0 to 1. In such experiments, it was found that increasing the bulk analyte concentration, $c_{0}$, resulted in an increase of ΔI_DS_($c_{0}$) with saturation at higher concentrations, $c_{\infty }$. The Langmuir model describes this relation between the coverage, Θ, and the bulk analyte concentration, $c_{0}$, by Equation (1):

(1) $$\Theta \left(c_{0}\right)=\frac{{\Delta I}_{\mathrm{DS}}\left(c_{0}\right)}{{\Delta I}_{\mathrm{DS}}\left(c_{\infty }\right)}=\frac{c_{0}/K_{d}}{\left(1+c_{0}/K_{d}\right)}$$

Alternatively, with the flow cell attached, one can measure the kinetics of the sensor response, ΔI_DS_, after changing the concentration in the bulk to another value. For an increase of the bulk concentration this can be described in the Langmuir model by Equation (2):

(2) $$\Theta \left(t\right)=\frac{c_{0}/K_{d}}{\left(1+c_{0}/K_{d}\right)}\cdot \left(1-e^{-\left(k_{\mathrm{on}}c_{0}+k_{\mathrm{off}}\right)t}\right)$$

Measuring this association of kinetics at different bulk concentrations then allows for the determination of the rate constants, k_on_ and k_off_.

In the dissociation phase, e.g., upon changing the bulk liquid from an odorant solution of a certain concentration to pure buffer, the time-dependent change of the sensor response is described by Equation (3):

(3) $$\Theta \left(t\right)=\frac{c_{0}/K_{d}}{\left(1+c_{0}/K_{d}\right)}\;\cdot {\;e}^{-k_{\mathrm{off}}\;\cdot \;t}$$

The analysis of the data according to Equation (3) leads to an additional determination of the dissociation rate constant, k_off_. According to the model, the dissociation constant, K_d_, is given by Equation (4):

(4) $$K_{d}=k_{\mathrm{off}}/k_{\mathrm{on}}$$

which then allows for an internal test of the applicability of the Langmuir model by comparing the dissociation constant, K_d_, determined by two very different measurements: on the one side, from a titration experiment that measures the coverage at equilibrium, free from any time dependent changes, whereas the kinetics give the same parameter by recording the sensor response before an equilibrium is reached. The difference, if any, of the values for the dissociation constants determined by the two approaches can be considered as an internal consistency test for the applicability of the Langmuir model for a quantitative description of what happens at the sensor surface. This test also provides evidence for a 1:1 complex between the PBP and the ligands.

## 3. Results

The aim of this work was to verify whether a protein such as an OBP retained its specificity of binding when covalently immobilized on the biosensor surface. Therefore, we selected seven volatile compounds to test with the protein, using the fluorescent displacement assay, and to challenge the biosensor. These compounds include the two pheromones of *B. mori*, bombykol and bombykal, and five general odorants, produced by plants and widely present in the environment: eugenol, linalool, isoamyl acetate, geraniol, and 2-isobutyl-3-methoxypyrazine. In addition, as proof-of-concept, we tested the behavior of the protein at pH 4 using bombykol as the ligand. In fact, it has been shown that in acidic conditions the binding capacity of PBP1 of *B. mori*, similar to that of several other insect OBPs, becomes much weaker, due to a major conformational change. The C-terminal segment, which is not structured at neutral pH, due to repulsions between negatively charged carboxy groups, folds into an α-helical domain in acidic conditions and enters the binding pocket, thus preventing the binding of external ligands [32].

### 3.1. Affinities of BmorPBP1 to Odorants and Pheromones in Solution

To evaluate the affinities of the *B. mori* PBP in solution, we adopted a competitive assay, where the strength of binding is related to the ability of the ligand in displacing the fluorescent probe. Therefore, we first measured the affinity of the fluorescent ligand 1-phenylnaphthylamine (Figure 1A), which was found to be 0.75 μM (SD: 0.08).

Then, we evaluated the affinities of the recombinant PBP1 of *B. mori* to the other ligands. Dissociation constants of 0.94 and 1.1 μM were obtained for bombykol and bombykal, respectively, in agreement with previous reports [31], where the two ligands were shown to efficiently and similarly displace the fluorescence probe 1-NPN at concentrations of 7 μM. Of the other ligands, only eugenol could displace the fluorescent probe with a good dissociation constant of 2.8 μM, while the other chemicals tested, geraniol, linalool, isoamyl acetate, and 2-isobutyl-3-methoxypyrazine, showed only poor binding (Figure 1B and Table 1).

### 3.2. Affinities of BmorPBP1 to Odorants and Pheromones Measured by an Electronic Sensor

**Figure 2:**
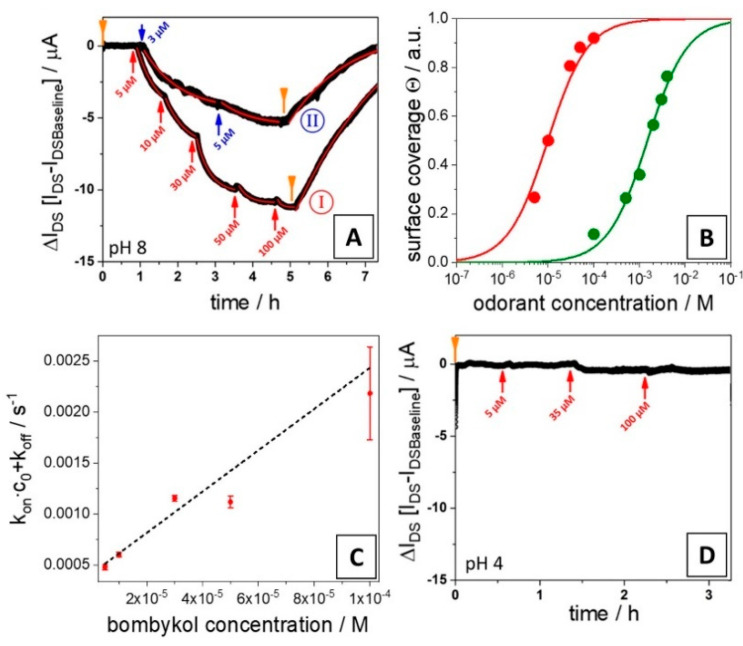
(**A**) Global analysis of the binding of bombykol (I) and bombykal (II) to a BmorPBP1-functionalized reduced graphene oxide field-effect transistors (rGO-FET) device, monitoring the association on the sensor surface for increasing analyte concentrations (3 μM to 100 μM) at pH 8, and the dissociation upon rising the flow cell with pure buffer; the red and the blue arrows indicate the addition of analyte solution of the given concentration, the orange ones mark the switching in the flow cell to pure buffer. The red curves in the data traces are the kinetic fits to the time-dependent changes, ΔI_DS_, of the source–drain current: with increasing concentration, this yields the binding rate constant, k_on_$c_{0}$ + k_off_, from the fit to the rinsing step one obtains k_off_ alone. (**B**) Langmuir isotherms, i.e., coverage, as a function of the analyte solution concentration, $c_{0}$: red full circles are the data points from bombykol (I) in (**A**), green full circles are from a corresponding measurement with 2-isobutyl-3-methoxypyrazine. The S-shaped curves are fits to the data; K_d_ -values thus obtained, K_d_ = 9.8 μM for bombykol, and 2.8 mM for 2-isobutyl-3-methoxypyrazine, respectively, are also summarized in Table 1. (**C**) k_on_$c_{0}$ + k_off_ values from the bombykol kinetic fits in **A** (I) as a function of the solution concentration; the slope is a linear fit to the data (according to Equation (2)) with k_on_= 20 M^−1^s^−1^; together with k_off_ = 9.4 10^−5^ s^−1^ from the dissociation curve, one obtains a kinetically-determined dissociation constant of K_d_ = 4.7 μM, in very good agreement with the value from the titration experiment in (**B**). (**D**) ΔI_DS_, monitored as a function of time after the addition of bombykol solutions of increasing concentration at pH 4; arrows as in (**A**).

Next, we measured the binding behavior of the same odorant ligands to the pheromone PBP1 from *Bombyx mori* with the electronic sensor. Figure 2 summarizes some of the measurements performed. The first example, given in Figure 2A, refers to the global analysis, i.e., a combination of a kinetic and a titration experiment, of the pheromones bombykol (I) and bombykal (II), to the protein receptor on the sensor surface: the association is measured as a function of time for various increasing bulk concentrations until, in each case, a new equilibrium value of the current is reached, followed by recording the dissociation kinetics, seen as the return of the current to the baseline.

The analysis of the data has two components: the first is the evaluation of the recordings of the sensor current, ΔI_DS_, after a new equilibrium is reached, as a function of the bulk concentration, $c_{0}$. Figure 2B shows the result for bombykol (I) in a lin-log plot (red full circles), together with the Langmuir fit to the data, which results in a value for the dissociation constant K_d_ = 9.8 μM. The excellent fit of the experimental data by the Langmuir model confirms the 1:1 complex formation between the protein and the ligand.

The alternative analysis, based on kinetic data taken for the different bulk concentrations of bombykol (cf. Figure 2A(I)) is given in Figure 2C. From the slope of the linear increase of the overall rate constant (k_on_$c_{0}$ + k_off_) as a function of $c_{0}$, we obtain a value for the association rate constant k_on_ = 20 M^−1^s^−1^. Together with the value for the dissociation rate constant k_off_ = 9.4 10^−5^ s^−1^, measured upon rinsing the sensor surface with pure buffer (cf. Figure 2A(I)), we can calculate a kinetically-determined dissociation constant K_d_ = 4.7 μM. In these types of measurements, a factor of 2 in the values obtained using the two different approaches is considered as an excellent agreement, confirming the validity of the application of the Langmuir model.

Next, the response of different ligands binding to the proteins on the electronic sensor device were tested. All data were taken by kinetic runs; in some cases, titration experiments were done in addition. Figure 2A(II) shows the kinetic data obtained from the other known pheromone of *B. mori*, i.e., bombykal. The analysis gives a dissociation constant of K_d_ = 7.4 μM, nearly identical to the value obtained for bombykol.

An example for a titration experiment for a low-affinity ligand, 2-isobutyl-3-methoxypyrazine, is given in Figure 2B (green full circles). As one can see, the fit to the data leads to a significantly reduced affinity, compared to bombykol, and yields a value of K_d_ = 2.8 mM. Analyzing the simultaneously taken corresponding kinetic data (not shown) results in a value for the dissociation constant of K_d_ = 1.3 mM, again in very good agreement with the value obtained from the titration experiment.

A most interesting result is presented in Figure 2D; it shows the sensor response (of the same chip as the one used in A) for the binding of bombykol; this time, however, the acidity of the analyte solution was shifted to pH 4. The feedback of the current is barely detectable, and only a lower limit of the affinity constant can be given. This change was fully reversible: switching back to a bombykol solution of pH 8 gave a (nearly) identical global analysis curve to the one presented in Figure 2A(I).

**Figure 3:**
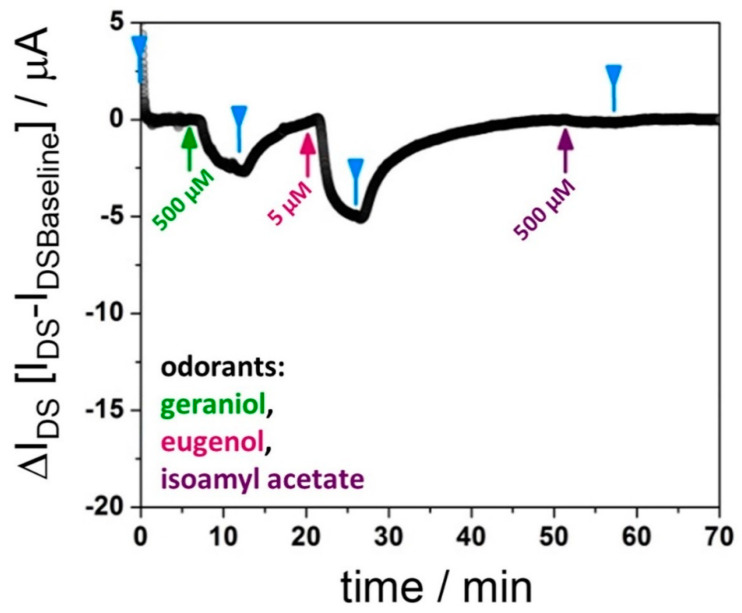
Kinetic runs with one rGO-FET device, functionalized with BmorPBP1; however, subsequently exposed in the flow cell to different analyte (odorant) solutions: geraniol, eugenol, isoamyl acetate, as indicated; binding (k_obs_) and dissociation (k_off_) rate constants are obtained from fits according to Equations (2) and (3), respectively, for geraniol and eugenol, and are used to approximate the dissociation constant for these ligands, as summarized in Table 1. Isoamyl acetate does not give a significant response of ΔI_DS_.

Another way of determining the relative binding affinities of different ligands is shown in Figure 3: here, the same chip is used for monitoring the association and dissociation events on the protein-functionalized sensor surface after injection of, firstly, 500 μM geraniol, followed by 5 μM eugenol, and then 500 μM isoamyl acetate. Even though the kinetic rate constants and, hence, the affinity constants, are based on a single kinetic run for each of the ligands, the fact that the same chip is used for the sequentially performed measurements excludes any uncertainties linked to differences in performance between sensor chips.

Table 1 summarizes all averaged results obtained for ligands investigated by the electronic sensor device, either by titration or by kinetic runs, and compares them with the data measured in solution.

## 4. Discussion

**Figure 4:**
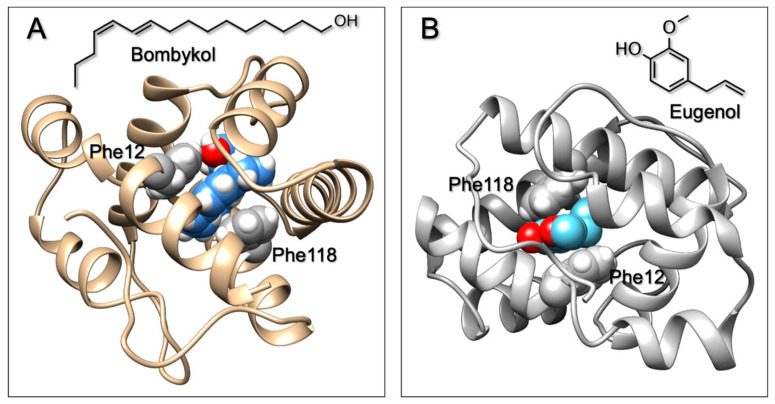
Docking simulations of the binding of bombykol (**A**) and eugenol (**B**) to the PBP1 of *B. mori*. In both cases, the molecule is sandwiched between two phenylalanine residues, Phe12 and Phe118. Good π−π interactions are established between the benzene rings of these two residues and the conjugated system of bombykol or the aromatic ring of eugenol. Docking simulation was performed using the online software Swiss Dock [40].

The amino acids lining the binding pocket of *B. mori* PBP1 and interacting with the ligand are: Ser56 (H-bond with OH of bombykol), Phe12 and Phe118 (two double bonds of bombykol sandwiched between these two residues), F36, Trp37, F76, plus a few hydrophobic residues, such as valine, leucine, and isoleucine [30]. Figure 4A shows the structure of the protein with a molecule of bombykol sandwiched between Phe12 and Phe118 [30]. Similar interactions might take place with the aromatic ligand eugenol, thus justifying the low values of dissociation constant measured for this chemical. This view is supported by docking simulations performed with eugenol (Figure 4B).

The solution behavior parallels the responses recorded from the sensor bearing the PBP immobilized via his-tag on a graphene layer, and indicates that the properties of the protein are not greatly affected by its immobilization. Thanks to the his-tag, the protein molecules are attached to the sensor all in the same direction, with the amino terminus closest to the graphene surface.

A comparison of the dissociation constants measured with the fluorescence assay in solution with the responses of the sensor (Table 1) shows a satisfactory correlation between results obtained by the two approaches. Even though the absolute values between the solution affinities and those found with the OBP immobilized on a sensor surface differ by up to as much as an order of magnitude (with the differences between the titration and the kinetic data alone accounting for an uncertainty of a factor 2), one finds that the general trend of the binding affinities for different ligands found in solution strictly parallels the trend determined by an electronic sensor. This allows to predict, within certain limits, the performance of a biosensor with reference to its selectivity from ligand-binding measurements in solution. This fact proves particularly useful when designing mutations to be introduced in the structure of an OBP in order to narrow or modify its selectivity. Often this is achieved by a process of trial-and-error, typically requiring more than a single attempt to obtain the desired results. During this phase, it could be enough to verify the binding properties of the modified protein in solution until the desired target will be achieved. Then, we can assume with a certain confidence that the engineered OBP would perform in a similar way when immobilized on a sensor.

A particularly remarkable result of the sensor data is the finding that the protein—despite being immobilized on the sensor surface—is able to undergo a reversible conformational reorganization that results, such as in solution, in a dramatic change of the binding affinity to bombykol. To our knowledge, this is the first time that the binding properties of a surface-attached receptor could be controlled by a reversible structural reorganization induced by a solution parameter, the pH in this case. Most likely, this is possible only because the coupling strategy via the his-tag at the N-terminus of the protein allows the other free side of the protein to undergo, in acidic conditions, the conformational changes by which the C-terminal segments fold into α-helical domains and enter the binding pocket, as proposed for the solution mechanism of the pH control of the ligand affinity of the protein [32].

The results presented here are only proof-of-concept data; a more detailed study is currently under way that explores general concepts for post-immobilization modifications of receptor proteins. Similar to other post-translational modifications of proteins, e.g., phosphorylation or glycosylation, pH-induced structural changes of proteins in solution can result in significant property changes. This is well-documented for enzymes; for odorant binding proteins, this has been identified only recently as a source for major property changes. For surface-immobilized binding proteins, this is a totally new phenomenon that might, however, result in very interesting control concepts for biomimetic smell sensors.

## Figures and Tables

**Table 1:** Dissociation constants evaluated from the sensor responses, and in solution with the fluorescent competitive assay. Bombykol, bombykal, and eugenol proved to be the best ligands with both methods. Noteworthy is also the loss of binding activity at pH 4, observed not only in solution, as reported in the literature [32], but also with the immobilized protein.

	Sensor Data		Solution Data
Ligand	K_d_/μM		K_d_/μM
	Titration	Kinetics	
Bombykol (pH 8.0)	9.8	8.2	0.94
Bombykol (pH 4.0)	-	>1000	>100
Bombykal	-	7.4	1.1
Eugenol	7.6	8.1	2.8
2-Isobutyl-3-methoxypyrazine	2800	1300	>100
Geraniol	-	470	~20
Linalool	-	>1000	~40
Isoamyl acetate	-	>1000	~20

## Data Availability

Data sharing not applicable.
